# Guanylate binding proteins facilitate caspase-11-dependent pyroptosis in response to type 3 secretion system-negative *Pseudomonas aeruginosa*

**DOI:** 10.1038/s41420-018-0068-z

**Published:** 2018-06-27

**Authors:** Arjun Balakrishnan, Rajendra Karki, Brent Berwin, Masahiro Yamamoto, Thirumala-Devi Kanneganti

**Affiliations:** 1 Department of Immunology, St. Jude Children’s Research Hospital, Memphis, TN 38105 USA; 2 Department of Microbiology and Immunology, Geisel School of Medicine at Dartmouth, Lebanon, NH 03756 USA; 3 Department of Immunoparasitology, Research Institute for Microbial Diseases, Laboratory of Immunoparasitology, World Premier International Immunology Frontier Research Center, Osaka University, 3-1 Yamadaoka, Suita, Osaka 565-0871 Japan

## Abstract

Detection of bacterial ligands is a pre-requisite for inflammasome activation. During *Pseudomonas aeruginosa* infection, flagellin which is secreted through the T3SS is detected by the NLRC4 inflammasome. Activation of the NLRC4 inflammasome is believed to contribute to high IL-1β production and pathogenicity in cystic fibrosis patients with chronic *P. aeruginosa* infection. Interestingly, the majority of *P. aeruginosa* isolated from cystic fibrosis patients with chronic airway infection are non-motile and T3SS-negative, suggesting that yet un-characterized inflammasome pathways regulate IL-1β production in cystic fibrosis patients. Here we demonstrate the role of guanylate-binding proteins (GBPs) in regulating bacterial proliferation and inflammasome activation in response to T3SS-negative *P. aeruginosa*. Bacterial ligands liberated by the action of GBP2 and IRGB10 activate caspase-11 and regulate non-canonical NLRP3 inflammasome activation and IL-1β release. Overall, our results reveal the role of caspase-11 in inhibiting bacterial proliferation and promoting IL-1β secretion during T3SS-negative *P. aeruginosa* infection. This study suggests that non canonical inflammasomes might have co-evolved to detect Gram-negative bacterial pathogens that have evolved to bypass detection by canonical NLRs.

## Introduction

*P. aeruginosa* is an extracellular opportunistic pathogen that is responsible for both acute and chronic infections in humans. *P. aeruginosa* can cause acute infection in patients with a variety of predisposing conditions such as immunodeficient subjects, burn victims and patients on mechanical ventilation^1^. *P. aeruginosa* can cause chronic airway infection and associated pulmonary damage in cystic fibrosis patients which leads to accumulation of pro-inflammatory cytokines including IL-1β in the sputum^2^. Even though detrimental to the host at high level, a low level of IL-1β has been shown to be beneficial for clearance of *P. aeruginosa* from the infected lungs^3,4^.

IL-1β secretion during bacterial infection is controlled by multimeric innate immune signaling complexes called inflammasomes^5^. During bacterial infection, bacterial ligands or host cell damage is sensed by the inflammasome sensors NLRP1, NLRP3, NLRC4, Pyrin, or AIM2. Recently, a new form of non-canonical NLRP3 inflammasome activation was described that is mediated by sensing of bacterial LPS by caspase-11^6,7^. Once activated, inflammasome sensors initiate the formation of an inflammasome complex containing ASC and caspase-1. Activated caspase-1 proteolytically processes inflammatory cytokines, pro-IL-1β and IL-18, and also activates a form of cell death called pyroptosis. Activation of inflammasomes thus restricts the dissemination of pathogen in two ways. Pyroptosis itself inhibits the spread of the pathogen by destructing the bacterial replication niche. In addition, secretion of inflammatory cytokines leads to the recruitment of immune cells that contain the pathogen at the site of infection^5^.

Flagellin is the monomer of flagellar apparatus associated with bacterial motility. During *P.aeruginosa* infection, flagellin is secreted through the T3SS into the cytosol of macrophage and is known to activate NLRC4 inflammasome^1^. Interestingly, majority of *P. aeruginosa* isolated from cystic fibrosis patients with chronic airway infection are either non-motile or are T3SS-negative^8–10^. These data suggest that NLRC4 inflammasome activation may not be the major contributor for IL-1β production during chronic airway infection seen in cystic fibrosis patients. This in turn indicates the activation of yet unidentified inflammasome sensors that detect the presence of *P. aeruginosa* and regulate the secretion of IL-1β. The above observations prompted us to investigate the role of innate immune sensors other than NLRC4 in mediating inflammasome activation during T3SS-negative *P*. aeruginosa infection.

In this study, we compared inflammasome activation by WT, non-motile and T3SS-negative *P. aeruginosa* and demonstrated for the first time that T3SS activates both NLRP3 and NLRC4 inflammasomes. The T3SS-negative *P. aeruginosa* failed to activate NLRC4 and NLRP3 inflammasomes during acute infection. However, T3SS-negative *P*. aeruginosa activated the non-canonical NLRP3 inflammasome during long term infection. We further demonstrate the requirement for interferon-inducible proteins IRGB10 and GBP2 in promoting non-canonical inflammasome activation. Additionally, GBP2 also regulates bacterial survival in macrophages. Overall, our findings help to explain the source of IL-1β secretion in cystic fibrosis patients with chronic airway infection due to *P. aeruginosa* where majority of the strains are T3SS-negative. The ability of GBPs in clearing T3SS-negative *P. aeruginosa* from macrophages also prompts the possibility of using interferons for activating cell autonomous immunity to promote clearance of *P. aeruginosa* from cystic fibrosis patients.

## Results

### T3SS mediates flagellin-independent inflammasome activation during *Pseudomonas* infection

**Fig. 1:**
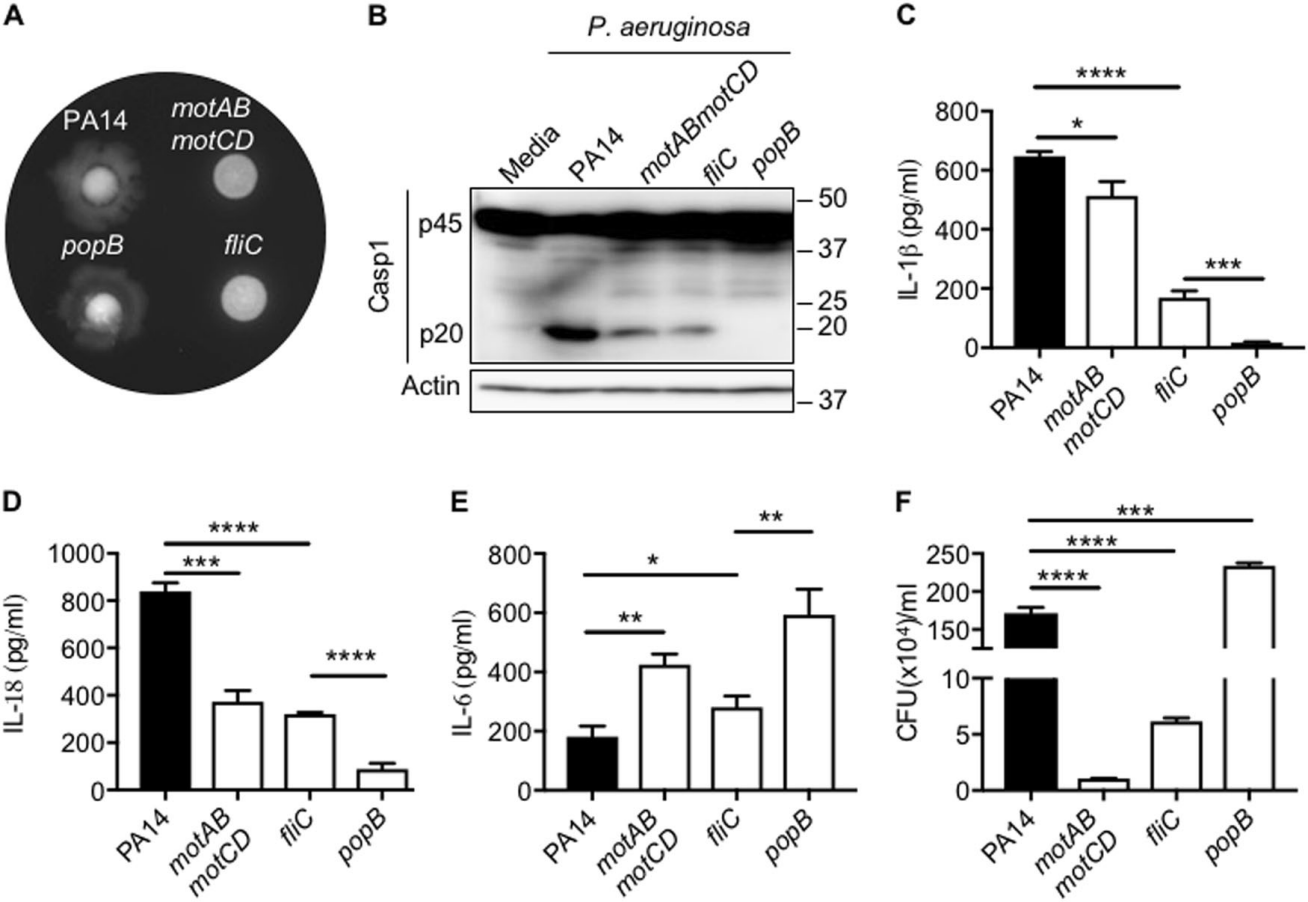
Non-motile *P. aeruginosa activates* caspase-1 through T3SS. **a** The motility phenotype of strains (PA14, *motAB motCD, fliC, popB*) confirmed in soft agar. **b**–**e** Caspase-1 activation, IL-1β, IL-18, and IL-6 release in unprimed bone marrow-derived macrophages (BMDMs) infected with indicated strains of *P. aeruginosa* (MOI 10) for 2 h. **f** Intracellular bacterial numbers in unprimed BMDMs infected with indicated strains of *P. aeruginosa* (MOI 1) for 30 min. Data are representative of two (**a**, **f**) or three (**b**–**e**) independent experiments. *p < 0.05, **p < 0.01, ***p < 0.001, ****p < 0.0001 (Two-tailed *T* test)

*P. aeruginosa* strains isolated from chronically infected cystic fibrosis patients have either defective T3SS or are non-motile (aflagellated or flagellated, but with defective motor apparatus)^8,9^. To understand which of these mutations give a preferential advantage over bacterial detection and activation of inflammasome, BMDMs (Bone marrow-derived macrophages) were infected with either WT bacteria (PA14), T3SS mutant (*popB*), aflagellated bacteria (*fliC*) or flagellated but non-motile bacteria (*motABmotCD*). The motility defects in these strains were confirmed by motility assay (Fig. 1a). A substantial reduction in caspase-1 activation was observed in BMDMs infected with *motABmotCD* and *fliC* compared to BMDMs infected with PA14 (Fig. 1b). We also observed significant reduction in secretion of inflammasome-dependent cytokines IL-1β and IL-18 in BMDMs infected with *motABmotCD* and *fliC* compared to PA14-infected BMDMs (Fig. 1c, d). These results demonstrate that motility plays a major role in mediating inflammasome activation during *P. aeruginosa* infection. Interestingly, inflammasome activation was completely absent in *popB*-infected BMDMs (Fig. 1b-d). Overall, this suggests that PAMPs (Pathogen-associated molecular patterns) that are secreted through the T3SS can also activate inflammasomes even in the absence of flagellin.

Inflammasome activation is a two-step process that requires priming by bacterial ligands or cytokines and activation by PAMPs or DAMPS. The differences in inflammasome activation during infection with different bacterial strains could be due to decreased bacterial uptake or decreased priming^11^. In order to distinguish these pathways, cells were infected with *P. aeruginosa* and bacterial uptake was quantified within 30 min of infection. Bacterial internalization was significantly less in *motABmotCD* and *fliC*-infected BMDMs as compared to PA14-infected BMDMs suggesting reduced phagocytosis of *motABmotCD* and *fliC* (Fig. 1f). Interestingly, IL-6 levels were slightly increased in *motABmotCD* and *fliC-*infected BMDMs (Fig. 1e). These results suggest that decreased inflammasome activation during *motABmotCD* and *fliC* infection could be due to decreased bacterial uptake, and is not due to decreased priming of inflammasome. The decreased inflammasome activation in *fliC* mutant could also be due to reduced secondary signal (flagellin) that is necessary for NLRC4 inflammasome activation. In contrast, bacterial uptake as well as IL-6 levels were high in *popB*-infected BMDMs compared to PA14-infected BMDMs. Therefore, the lack of inflammasome activation during *popB* infection indicates the requirement for an active T3SS to secrete flagellin and other unidentified factors that facilitate inflammasome assembly and activation.

### T3SS-mediated inflammasome activation leads to formation of single ASC foci containing caspase-1 and caspase-8

**Fig. 2:**
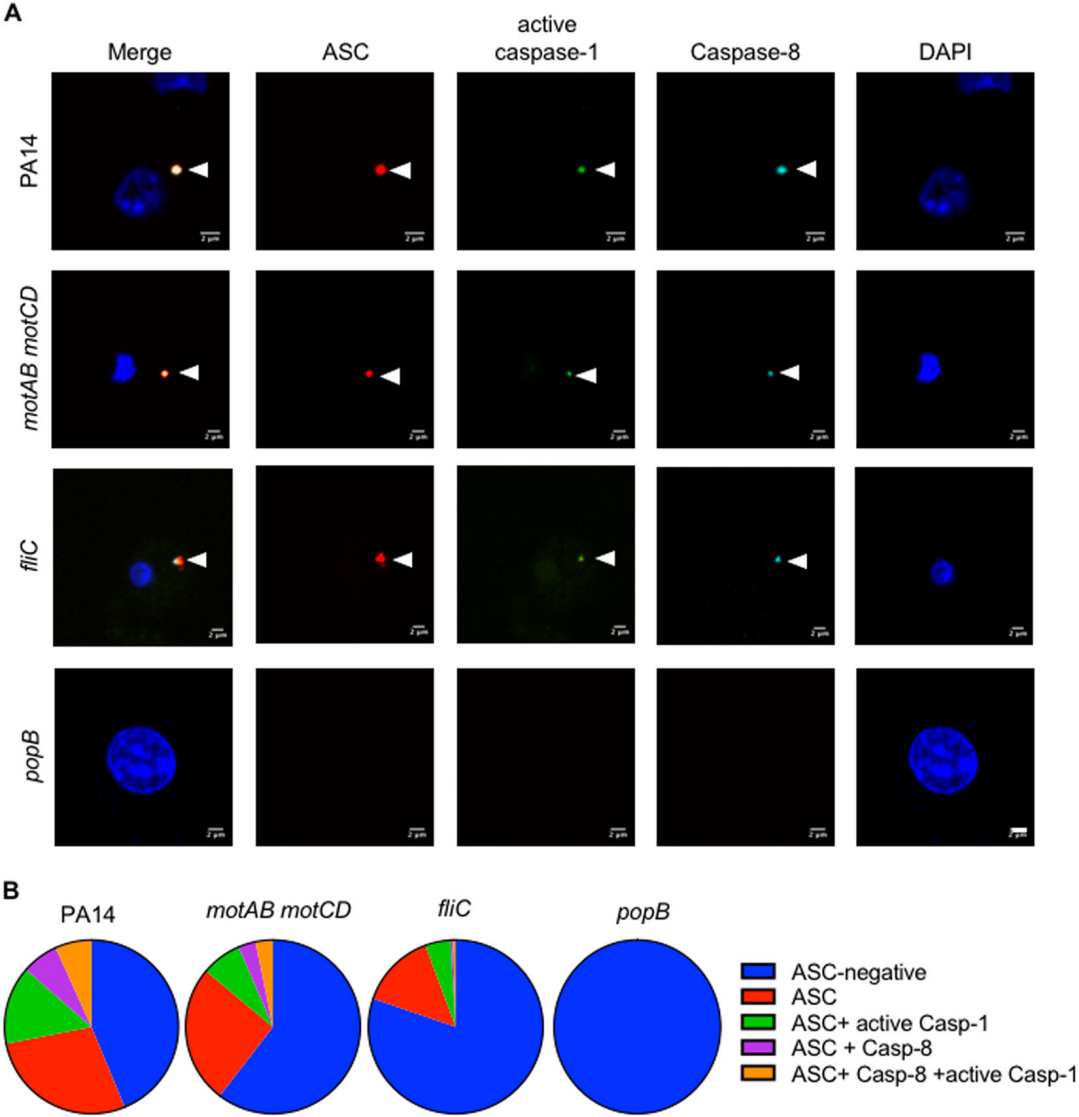
ASC foci formation in macrophages by *P. aeruginosa* is T3SS dependent. **a** Unprimed BMDMs were infected with indicated strains of *P. aeruginosa* (MOI 10) for 1 h and stained for ASC (red), active caspase-1 (green), caspase-8 (magenta) and DNA (blue). Arrow heads indicate an inflammasome complex. (Scale, 2 μm). **b** Composition of ASC specks. At least 200 BMDMs infected with indicated strains were counted

In order to investigate whether inflammasome assembly is impaired during T3SS mutant infection, we assessed the formation of “ASC specks” in the infected cells. “ASC specks” or pyroptosomes are complex multimeric protein structures formed in response to inflammasome activating stimuli^12^. These structures contain the inflammasome sensor, ASC and caspase-1. Recruitment of caspase-8 to the pyroptosome was reported during *Salmonella* and *Aspergillus* infection^13,14^. Immunofluorescent microscopy of PA14-infected BMDMs revealed formation of ASC foci containing both active caspase-1 and caspase-8 (Fig. 2a). The composition of pyroptosome and number of pyroptosomes varied among cells infected with different mutants (Fig. 2a, b). The number of ASC foci and ASC foci containing active caspase-1 was significantly less in *fliC*-infected BMDMs compared to PA14-infected BMDMs suggesting a key role of flagellin in mediating inflammasome assembly. Despite high bacterial numbers in the cytoplasm during *popB* infection, there was no detectable ASC foci in the cell (Fig. 1f, Fig. 2a, b). This further demonstrates that activity of T3SS is necessary for promoting inflammasome assembly.

### *P. aeruginosa* T3SS activates NLRC4 and NLRP3 inflammasomes

**Fig. 3:**
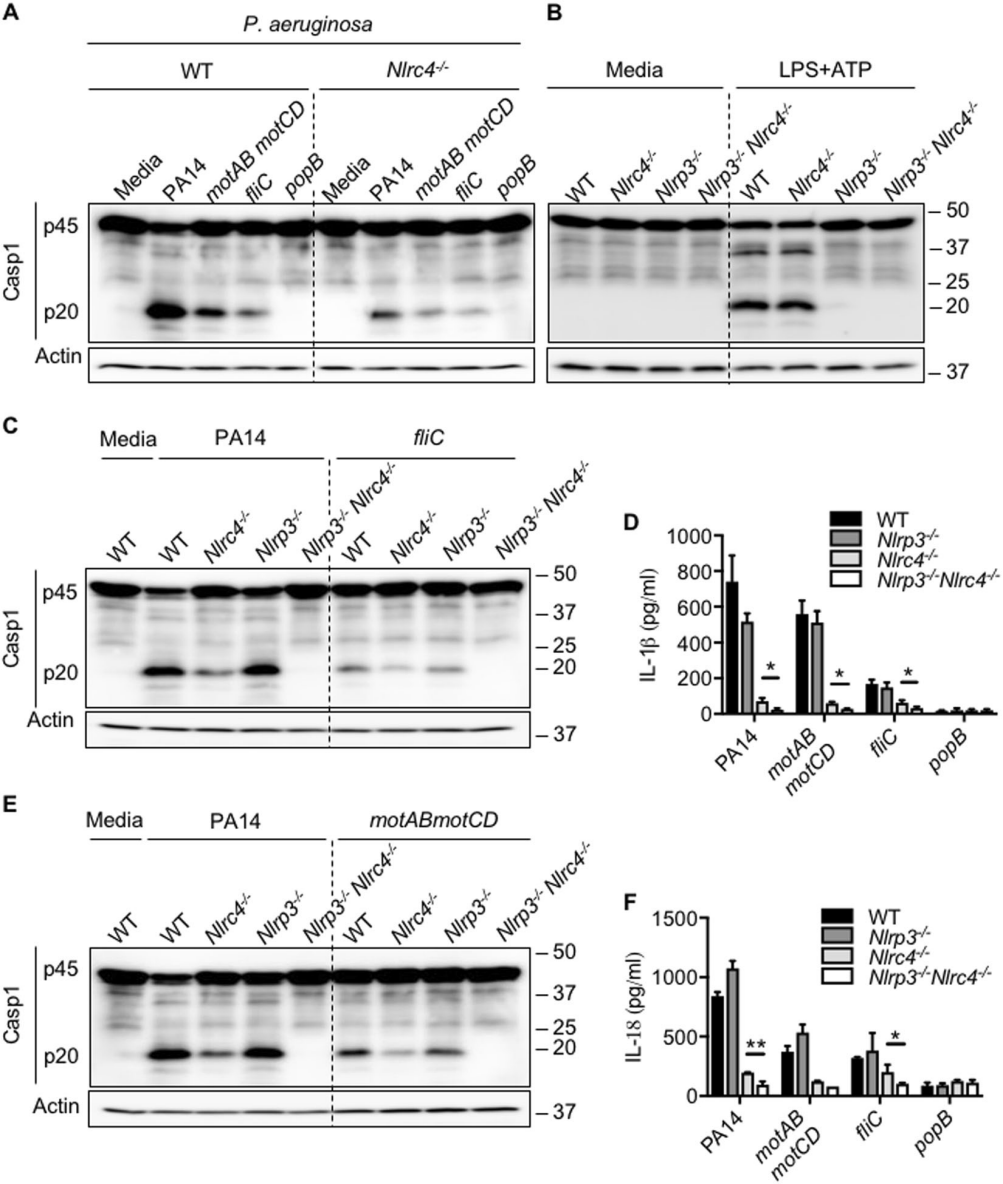
*Pseudomonas aeruginosa* T3SS induces NLRP3 inflammasome in the absence of NLRC4 inflammasome. **a** Immunoblot analysis of caspase-1 in unprimed WT, *Nlrc4*^−*/*−^ BMDMs left untreated or infected with indicated strains of *P. aeruginosa* (MOI 10) for 2 h. **b** Immunoblot analysis of caspase-1 in unprimed or LPS-primed WT, *Nlrc4*^−*/*−^*, Nlrp3*^−*/*−^ and *Nlrp3*^−*/*−^*Nlrc4*^−*/*−^ BMDMs stimulated with ATP (LPS + ATP). **c**–**f** Immunoblot analysis of caspase-1, IL-1β, and IL-18 release in unprimed WT, *Nlrc4*^−*/*−^*, Nlrp3*^−*/*−^ and *Nlrp3*^−*/*−^*Nlrc4*^−*/*−^ BMDMs left untreated or infected with indicated strains of *P. aeruginosa* (MOI 10) for 2 h. Data are representative of three independent experiments. *p < 0.05, **p < 0.01 (two-tailed *T* test)

During *P. aeruginosa* infection, flagellin secreted through the T3SS is sensed by NLRC4 and leads to inflammasome activation^15^. To understand whether inflammasome activation in *fliC* mutant is also dependent on NLRC4, we infected WT and *Nlrc4*^−*/−*^ BMDMs with PA14, *fliC, motABmotCD*, and *popB*. Interestingly, inflammasome activation and caspase-1 cleavage is observed even in the absence of NLRC4 during *P. aeruginosa* infection albeit at reduced levels (Fig. 3a). The inflammasome activation in the absence of NLRC4 during *P. aeruginosa* infection is not strain-dependent as the same was observed during infection with *P. aeruginosa* strains PA01 and PAK (Fig S1A-C).

NLRP3 is known to be associated with NLRC4 during *Salmonella* infection and regulate inflammasome activation^16^. We hypothesized that the residual inflammasome activation during *P. aeruginosa* infection is through activation of NLRP3 inflammasome. To validate this hypothesis, we infected *Nlrp3*^−*/*−^ and *Nlrp3*^−*/*−^*Nlrc4*^−*/*−^ BMDMs with PA14. There was no significant difference in cas*3*^−*/*−^pase-1 cleavage or secretion of IL-1β and IL-18 in *Nlrp3*^−*/*−^ BMDMs compared to WT BMDMs (Fig. 3c, d, f). Under similar conditions, activation of canonical NLRP3 inflammasome by LPS and ATP is completely abrogated in *Nlrp3*^−*/*−^ BMDMs (Fig. 3b). Interestingly, caspase-1 cleavage during *P. aeruginosa infection* was completely abrogated in *Nlrp3*^−*/*−^*Nlrc4*^−*/*−^ BMDMs (Fig. 3c). We also observed complete reduction in IL-1β and IL-18 secretion in *Nlrp3*^−*/*−^*Nlrc3*^−*/*−^ BMDMs infected with PA14 suggesting that NLRP3 mediates inflammasome activation in the absence of NLRC4 during *P. aeruginosa* infection (Fig. 3d, f). To further investigate whether flagellin is solely responsible for inflammasome activation during *P. aeruginosa* infection, we infected WT, *Nlrp3*^−*/*−^ and *Nlrc4*^−*/*−^ BMDMs with *fliC* mutant. We observed reduced caspase-1 cleavage, IL-1β, and IL-18 secretion in both NLRP3 and NLRC4-deficient BMDMs infected with *fliC* suggesting that factors other than flagellin can facilitate both NLRP3 and NLRC4 inflammasome activation during *P. aeruginosa* infection (Fig. 3c, d, f). Inflammasome activation in *motABmotCD-*infected BMDMs was also dependent upon NLRP3, confirming that non-motile bacteria can activate NLRP3-dependent inflammasomes (Fig. 3d–f). Interestingly, inflammasome activation was completely abrogated in BMDMs in response to *popB* infection (Fig. 3a). This suggests that T3SS mediates activation of both NLRP3 and NLRC4 inflammasomes during *P. aeruginosa* infection.

To understand whether NLRP3 inflammasome activation regulates pyroptosome formation in the cytoplasm during *P. aeruginosa* infection, we quantified the ASC foci and components of ASC foci in BMDMs infected with PA14 (Fig S2A). Even though there was no decrease in ASC foci formation in *Nlrp3*^−*/*−^ BMDMs, the number of ASC foci containing active caspase-1 and caspase-8 was less in *Nlrp3*^−*/*−^ BMDMs compared to WT BMDMs (Fig S2B). Similar to inflammasome activation in *Nlrc4*^−*/*−^ BMDMs, pyroptosomes were observed in *Nlrc4*^−*/*−^ BMDMs confirming that cells undergo NLRP3-dependent pyroptosis in the absence of NLRC4 during *P. aeruginosa* infection (Fig S2A,B). Although caspase-8 is recruited to the inflammasome complex, lack of caspase-8 did not affect inflammasome activation during *P. aeruginosa* infection as evidenced by similar caspase-1 cleavage in WT, *Ripk3*^−*/*−^ and *Ripk3*^−*/*−^*Cas3*^−*/*−^ BMDMs infected with PA14 (Fig S2C). To confirm the importance of ASC in inflammasome activation during *P. aeruginosa* infection, we infected *Asc*^−*/*−^ BMDMs with PA14. *P. aeruginosa* mediated caspase-1 cleavage in BMDMs was completely dependent on ASC (Fig S2D). Overall, these results suggest that T3SS activates both NLRP3 and NLRC4 inflammasomes during *P. aeruginosa* infection.

### Caspase-11-dependent pyroptosis inhibits formation of a replicative niche by T3SS mutant in macrophages

**Fig. 4:**
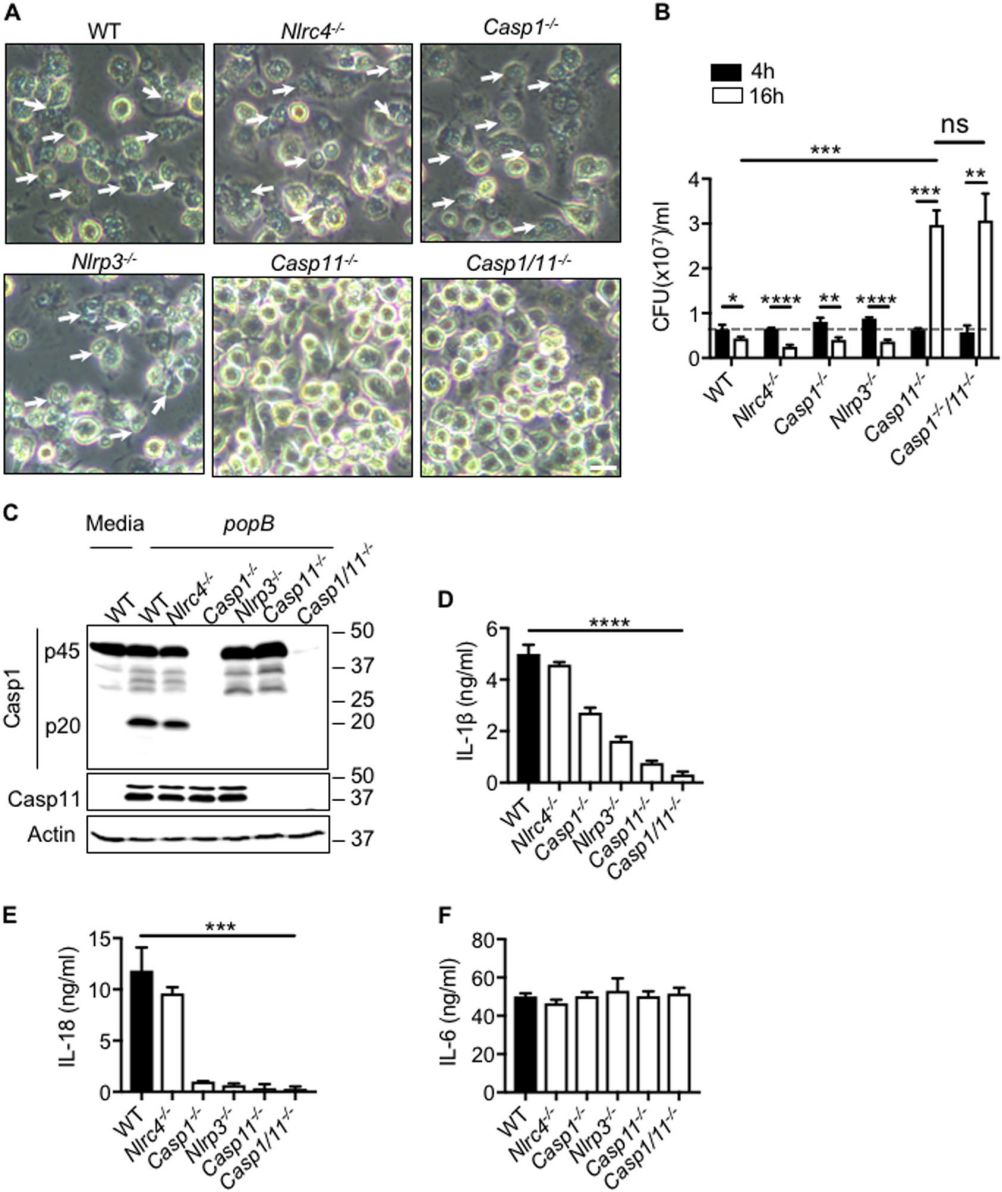
Caspase-11 mediated pyroptosis restrict proliferation of T3SS mutant in macrophages. **a** Microscopic analysis of cell death in unprimed WT, *Nlrc4*^−*/*−^*, Caspase1*^−*/*−^*, Nlrp3*^−*/*−^*,Caspase11*^−*/*−^ and *Caspase1*^−*/*−^ *Caspase11*^−*/*−^ BMDMs infected with *popB* (MOI 10) for 16 h. (Scale, 15 μm). Arrow heads indicate pyroptotic cells. **b** Bacterial CFU in unprimed WT, *Nlrc4*^−*/*−^, *Caspase1*^−*/*−^, *Nlrp3*^−*/*−^, *Caspase11*^−*/*−^ and *Caspase1*^−*/*−^ *Caspase11*^−*/*−^ BMDMs infected with *popB* MOI10, 4 h and 16 h. **c-f** Immunoblot analysis of caspase-1, IL-1β, IL-18, and IL-6 release in unprimed WT, *Nlrc4*^−*/*−^, *Caspase1*^−*/*−^*, Nlrp3*^−*/*−^*, Caspase11*^−*/*−^, and *Caspase1*^−*/*−^ *Caspase11*^−*/*−^ BMDMs left untreated or infected with *popB* for 16 h. Data are representative of two (**b**) or three (**a**,**c**–**f**) independent experiments. ns not significant, *p < 0.05, **p < 0.01, ***p < 0.001, ****p < 0.0001 (two-tailed *T* test)

*P. aeruginosa* T3SS mutants are associated with chronic airway infection^8^. We investigated whether the inability of the mutant to activate NLRC4 and NLRP3 inflammasome allows the mutant to form a replicative niche in macrophages. To address this question, we infected BMDMs with *popB* and bacterial numbers were quantified 4 h and 16 h post infection. Interestingly, we observed significant amount of pyroptosis in WT BMDMs during later time points of infection and bacteria were not able to proliferate inside macrophages (Fig. 4a, b). To understand whether NLRP3 and NLRC4 inflammasomes induce pyroptosis during chronic infection by *popB*, *Nlrp3*^−*/*−^ and *Nlrc4*^−*/*−^ BMDMs were infected with *popB* and caspase-1 cleavage was quantified (Fig. 4c). *Nlrc4*^−*/*−^ BMDMs showed similar levels of caspase-1 cleavage and pyroptosis compared to WT BMDMs (Fig. 4a, c). IL-1β and IL-18 secretion and bacterial survival was similar in *Nlrc4*^−*/*−^ and WT BMDMs (Fig. 4a–e). These results suggest that NLRC4 does not regulate pyroptosis during long term infection with *popB*. *Nlrp3*^−*/*−^ BMDMs also showed significant amount of pyroptosis during *popB* infection (Fig. 4a). Interestingly, caspase-1 cleavage and secretion of inflammasome dependent cytokines IL-1β and IL-18 was completely abrogated in *Nlrp3*^−*/*−^ BMDMs (Fig. 4c–e). These results point to caspase-11 dependent activation of non-canonical NLRP3 inflammasome in *popB-*infected BMDMs. In order to confirm this hypothesis, we infected *caspase-11*^−*/*−^ BMDMs with *popB* and quantified caspase-1 cleavage, IL-1β and IL-18 secretion. Pyroptosis, caspase-1 cleavage and secretion of inflammasome dependent cytokines IL-1β and IL-18 were completely abrogated in *caspase-11*^−*/*−^ BMDMs (Fig. 4a,c-e). IL-6 levels did not reduce in any of these conditions suggesting that the absence of inflammasome activation in *caspase-11*^−*/*−^ BMDMs is not due to a defect in priming of inflammasome (Fig. 4f). These results suggest that *popB* activates caspase-11-dependent non-canonical NLRP3 inflammasome during chronic infection.

During bacterial infection, caspase-1 and caspase-11 are known to inhibit bacterial proliferation independent of pyroptosis^17^. To understand whether caspase-11 mediated activation of caspase-1 through non-canonical NLRP3 inflammasomes regulate bacterial growth, we compared bacterial growth in WT, *Nlrp3*^−*/*−^, *Nlrc4*^−*/*−^*, caspase-1*^−*/*−^, *caspase-11*^−*/*−^ and *caspase-1*^−*/*−^*caspase-11* BMDMs (Fig. 4b). Presence or absence of caspase-1 did not significantly affect survival of *popB* in BMDMs. Surprisingly, absence of caspase-11 led to a bacterial proliferation in macrophages suggesting that caspase-11 regulates bacterial proliferation independent of caspase-1 activation during *popB* infection (Fig. 4b).

### GBP2 and IRGB10 facilitate caspase-11 activation during *popB* infection

**Fig. 5:**
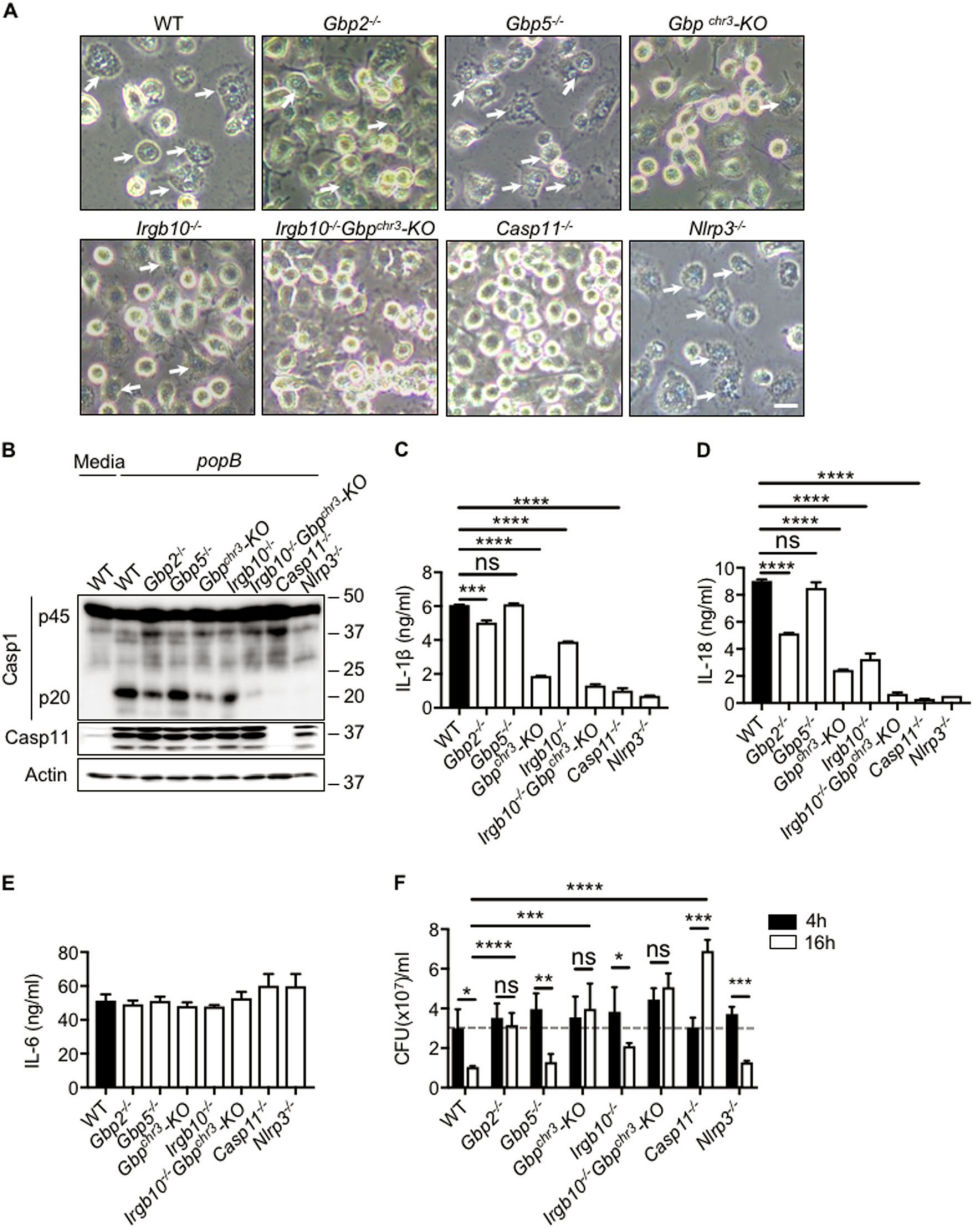
GBP2 and IRGB10 regulates caspase-11 mediated non-canonical NLRP3 activation and pyroptosis. **a** Microscopic analysis of cell death in unprimed WT, *Gbp2*^−*/*−^*, Gbp5*^−*/*−^*, Gbp*^*chr3*^-KO*,Irgb10*^−*/*−^*, Irgb10*^−*/*−^ *Gbp*^*chr3*^-KO, *Caspase11*^−*/*−^ and *Nlrp3*^−*/*−^ BMDMs infected with *popB* (MOI 10) for 16 h. (Scale, 15 μm). Arrow heads indicate pyroptotic cells. **b**–**e** Immunoblot analysis of caspase-1, IL-1β, IL-18, and IL-6 release in WT, *Gbp2*^−*/*−^*, Gbp5*^−*/*−^*, Gbp*^*chr3*^-KO*, Irgb10*^−*/*−^*, Irgb10*^−*/*−^ *Gbp*^*chr3*^-KO, *Caspase11*^−*/*−^ and *Nlrp3*^−*/*−^ BMDMs infected with *popB* (MOI10). **f** Bacterial CFU in unprimed WT, *Gbp2*^−*/*−^*, Gbp5*^−*/*−^*, Gbp*^*chr3*^-KO*, Irgb10*^−*/*−^*, Irgb10*^−*/*−^ *Gbp*^*chr3*^-KO, *Caspase11*^−*/*−^ and *Nlrp3*^−*/*−^ BMDMs infected with *popB* (MOI10), 4 h and 16 h. Data are representative of three independent experiments. ns-not significant, *p < 0.05, **p < 0.01, ***p < 0.001,****p < 0.0001 (two-tailed *T* test)

Liberation of bacterial ligands by GBPs and IRGB10 are known to regulate non-canonical inflammasome activation during Gram-negative bacterial infections^18–20^. To understand whether GBPs and IRGB10 are involved in activation of caspase-11 during *popB* infection, *Gbp2*^−*/*−^, *Gbp5*^−*/*−^, *Gbp*^*chr3*^-KO and *Irgb10*^−*/*−^ BMDMs were infected with *popB*. Interestingly, pyroptotic cell death, visualized by microscopy and quantified by Sytox green uptake was significantly reduced in both *Gbp*^*chr3*^-KO and *Irgb10*^−*/*−^ BMDMs (Fig. 5a, Fig S3). Among GBPs, only GBP2 played a major role in regulating pyroptosis during *popB* infection and GBP5 was completely dispensable to regulate cell death (Fig. 5a, b, Fig S3). *Gbp2*^−*/*−^, *Gbp*^*chr3*^-KO and *Irgb10*^−*/*−^ also showed reduced caspase-1 cleavage and IL-1β and IL-18 release during *popB* infection (Fig. 5b–d). Caspase-1 cleavage, pyroptosis and release of pro-inflammatory cytokines IL-1β and IL-18 was completely abrogated in *Irgb10*^−*/*−^ *Gbp*^*chr3*^-KO BMDMs, suggesting that GBPs and IRGB10 coordinately regulate caspase-11 activation during *popB* infection (Fig. 5a–d). Under similar conditions, secretion of IL-6 was not affected suggesting that GBPs do not affect priming of inflammasomes during *popB* infection (Fig. 5e). As expected, GBPs and IRGB10 did not regulate caspase-1 cleavage during *Pseudomonas* PA14 infection (Fig S1D). These results suggest that despite high bacterial uptake and bacterial burden in the cytosol compared to PA14, *popB* mediated inflammasome activation still requires liberation of bacterial ligands by GBPs and IRGB10.

GBPs are known to inhibit bacterial replication independent of inflammasome activation^21,22^. To understand whether GBPs contain *popB* replication in macrophages, *Gbp2*^−*/*−^, *Gbp5*^−*/*−^, *Gbp*^*chr3*^-KO and *Irgb10*^−*/*−^ BMDMs were infected with *popB* and intracellular bacterial numbers were quantified 4 h and 16 h post infection. As shown before, *popB* cannot survive in WT BMDMs (Fig. 5f). However *popB* was able to survive in *Gbp2*^−*/*−^ BMDMs, suggesting that either GBP2 act as an anti-bacterial factor or can expose *popB* to other anti-bacterial molecules in the cytosol (Fig. 5f). Compared to WT BMDMs, there was no significant difference in survival of *popB* in *Gbp5*^−*/*−^ and *Irgb10*^−*/*−^ BMDMs suggesting a unique role for GBP2 in controlling *popB* proliferation in macrophages (Fig. 5f). These results suggest that even though GBP2 and IRGB10 release *popB* ligands leading to activation of caspase-11, GBP2 alone act as an anti-bacterial agent against *popB* in macrophages. Interestingly, despite having similar levels of pyroptosis in *Irgb10*^−*/*−^ *Gbp*^*chr3*^-KO compared to *caspase-11*^−*/*−^ BMDMs, popB replication was significantly high in caspase11^−*/*−^ BMDMs (Fig. 5a–f, Fig S3). These results suggest that caspase-11 regulates bacterial proliferation independent of pyroptosis. Collectively, these results demonstrate that GBP2 and IRGB10 regulates caspase-11 activation and replication of *Pseudomonas T3SS mutants (popB)* which otherwise escape detection by NLRC4 and canonical NLRP3 inflammasomes. These findings point out to the importance of caspase-11 in inducing IL-1β secretion in cystic fibrosis patients chronically infected with T3SS-negative *P.aeruginosa*.

## Discussion

**Fig. 6:**
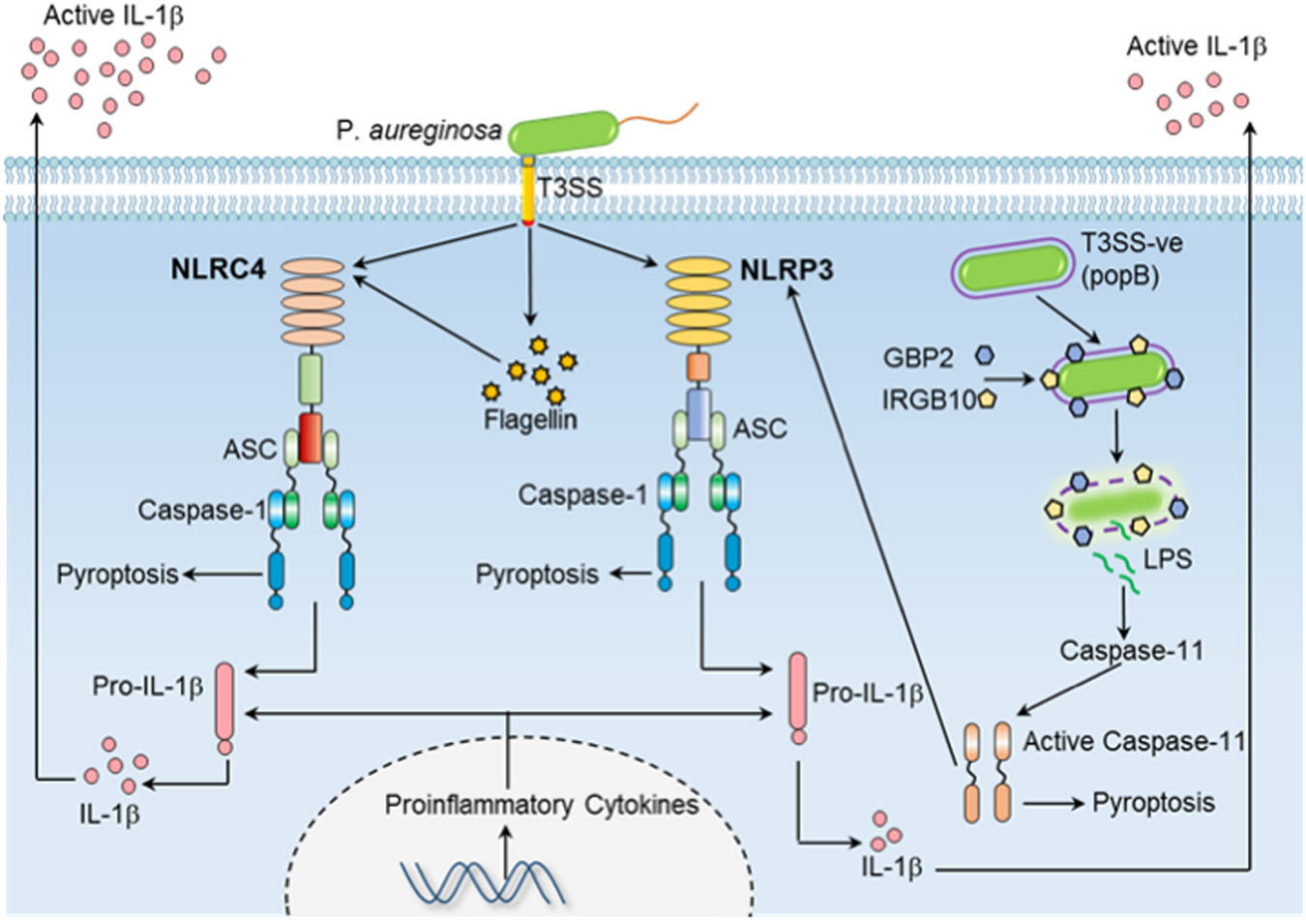
Model depicting inflammasome activation by WT and T3SS-negative *P.aeruginosa* during acute and chronic infection in macrophages. During acute infection (2 h), bacterial T3SS itself and flagellin secreted through T3SS activates NLRC4 inflammasome. T3SS also activates NLRP3 inflammasome by yet uncharacterized mechanisms. T3SS-negative *P.aeruginosa* escapes detection by both NLRP3 and NLRC4 inflammasomes and proliferates inside cytoplasm. During chronic infection (16 h), high numbers of T3SS-negative *P.aeruginosa* is detected by GBP2 and IRGB10. Bacterial lysis by action of GBPs release ligands that lead to activation of caspase-11. Caspase-11 inhibits the proliferation of bacteria and also activates NLRP3 inflammasome which leads to secretion of inflammatory cytokine IL-1β

In the present study, we showed that *P. aeruginosa* infection leads to activation of both NLRC4 and canonical NLRP3 inflammasomes (Fig. 6). Multiple inflammasomes are known to be activated simultaneously during bacterial and fungal infections^14,16^. Activation of multiple inflammasomes might help to give robust inflammasome activation under conditions where a specific inflammasome component is defective or is absent in a particular cell or tissue. In our study, we have detected robust NLRP3 inflammasome activation only in *Nlrc4*^−*/*−^ BMDMs. Under normal conditions, high activity of NLRC4 inflammasomes mask the role of NLRP3 inflammasomes during *P.aeruginosa* infection. Indeed, previous studies have demonstrated NLRP3 inflammasome activation in response to *Pseudomonas* infection in human bronchial epithelial cells derived from cystic fibrosis patients^23^. However, the expression of NLRC4 inflammasome in these cells and the PAMPs that can activate NLRP3 inflammasome are not well characterized.

NLRP3 inflammasome activation in flagellin-deficient *P.aeruginosa* demonstrates that flagellin is not the PAMP for NLRP3 inflammasome activation. NLRP3 inflammasome activation is completely abrogated during acute infection (2 h) with T3SS mutant *Pseudomonas*. T3SS needle apparatus is known to create pores on cell membrane^24^. Pore formation during bacterial infection can activate NLRP3 inflammasome^25^. Whether T3SS itself or the secreted virulence factors are mediating NLRP3 inflammasome activation is yet to be established. Components of T3SS needle apparatus are known to activate NLRC4 inflammasome^26^. T3SS needle apparatus might have contributed to residual NLRC4 inflammasome activation in *fliC-*infected *Nlrp3*^−*/*−^ BMDMs.

*P. aeruginosa* uses type 3 secretion system (T3SS) to inject T3SS effectors ExoU and ExoS to inhibit inflammasome activation^27^. These effectors inhibit caspase-1 activation by unknown mechanisms. Therefore, during *P. aeruginosa* infection, T3SS plays a dual role as inhibitor and activator of inflammasomes^28^. Theoretically, during chronic airway infection, evolution to reduce inflammasome activation should have selected *P. aeruginosa* strains that are defective in inducing inflammasome activation (Non-motile but T3SS +ve strains). Interestingly, 39% of *P. aeruginosa* isolated from cystic fibrosis patients with chronic airway infection are non-motile and 88% are T3SS negative^8,9^. These data suggest that T3SS itself induce inflammasome activation independent of flagellin or might have other inflammatory functions independent of inflammasome. The paradox of evolution of T3SS mutants (T3SS-ve) instead of non-motile but T3SS+ve strains during *P.aeruginosa* infection is not clear. Our study demonstrates that the evolution of T3SS mutants might be to help the bacteria to escape detection by both NLRP3 and NLRC4 inflammasomes (Fig. 6).

*P.aeruginosa* is known to cause chronic airway infections in cystic fibrosis patients. Even though majority of the strains isolated from these patients have defective T3SS, these patients still have significant amount of IL-1β in the sputum^2^. This suggests that during chronic airway infection, yet unknown PAMPs can contribute to inflammasome activation. Our study demonstrates that T3SS mutant *Pseudomonas* can be detected by IRGB10 and GBP2 (Fig. 6). Both GBP2 and IRGB10 are known to liberate ligands from bacteria^18^. Here, we demonstrated that during *popB* infection, only GBP2 contribute to bacterial killing whereas both GBP2 and IRGB10 were required to regulate inflammasome activation and cell death. This is different from inflammasome activation by *Citrobacter* or *Fransicella*, where the GBPs that regulate release of bacterial ligands can also regulate bacterial proliferation^19,22^. These data suggest that during *popB* infection, bacterial killing and generation of bacterial ligands are two separate processes. During *Fransicella* infection, GBP5 along with GBP2 and IRGB10 are important for activating AIM2 inflammasome^18^. Interestingly, GBP5 is completely dispensable for inflammasome activation during *popB* infection. These data further suggest that different GBPs can differentiate between different pathogens.

LPS liberated into the cytosol during Gram-negative bacterial infection is known to activate caspase-11^6,7^. During *popB* infection, we believe that LPS liberated by action of GBP2 and IRGB10 activates caspase-11-dependent non-canonical inflammasomes (Fig. 6). Interestingly, neither GBP2 nor IRGB10 is involved in >regulating inflammasome activation during infection with wild type *Pseudomonas*. Our study demonstrates an unidentified role of GBPs in regulating inflammasome activation during *Pseudomonas* infection. The fact that GBPs are exclusively relevant in inducing immune response to T3SS-negative *P.aeruginosa* demonstrates the fact that these mechanisms have evolved to detect pathogens that escape detection by canonical inflammasomes.

## Materials and methods

### Mice

C57BL/6J mice (wide-type [WT]) were purchased from The Jackson Laboratory and bred at St. Jude Children’s Research Hospital (SJCRH). *Nlrc4*^−*/*−^, *Nlrp3*^−*/*−^, *Caspase1*^−*/*−^*, Caspase11*^−*/*−^*, Caspase1*^−*/*−^*Caspase11*^−*/*−^*, Gbp2*^−*/*−^*, Gbp5*^−*/*−^*,Gbp*^*chr3*^-KO*, Irgb10*^−*/*−^*, Irgb10*^−*/*−^*Gbp*^*chr3*^-KO*,Rip3*^−*/*−^ *and Rip3*^−*/*−^*Cas8*^−*/*−^ were generated as described previously^14,18^. *Nlrp3*^−*/*−^*Nlrc4*^−*/*−^ mice were generated by crossing *Nlrp3*^−*/*−^ with *Nlrc4*^−*/*−^ mice. All mice were bred at the Animal Resource Center at SJCRH, and animal studies were conducted according to protocols approved by the SJCRH Animal Care and Use Committee.

### Bone marrow-derived macrophages

Primary bone marrow–derived macrophages (BMDMs) were grown for 5–6 days in DMEM (11995073, ThermoFisher Scientific) supplemented with 10% FBS (TMS-013-B, Millipore), 30% L929 conditioned media and 1% penicillin and streptomycin (15070–063, ThermoFisher Scientific). BMDMs were seeded in antibiotic-free media at a concentration of 1 × 10^6^ cells onto 12-well plates and incubated overnight.

### Bacterial strains and growth conditions

The *Pseudomonas aeruginosa* strain PA14 is a non mucoidal clinical isolate and is the parental strain for all the mutants studied. The *Pseudomonas aeruginosa* strain PAK, PAO1, PA14 and isogenic mutants of PA14 (*popB*, *fliC* and *motABmotCD*) were provided by Brent Berwin (Department of Microbiology and Immunology, Geisel School of Medicine at Dartmouth, Lebanon). Bacteria were inoculated in Luria–Bertani media (3002–031, MP Biomedicals) and incubated overnight under aerobic conditions at 37 °C. Bacteria were sub cultured (1:25) into fresh LB media for 2 1/2 h at 37 °C to generate log-phase bacteria for infection.

### Stimulation of bone marrow-derived macrophages

For acute bacterial infections, BMDMs were infected with indicated bacteria at an MOI of 10 for 2 h. For chronic infection with *popB*, BMDMs were infected at an MOI of 10 for 4 h. Cells were washed twice with PBS and were treated with 100 μg/ml gentamicin (15750–060, ThermoFisher Scientific) for 1 h to kill the extracellular bacteria. Cells were maintained in DMEM containing 10 μg/ml of gentamicin till 16 h. To activate the canonical NLRP3 inflammasome, BMDMs were primed using 500 ng/ml ultrapure LPS from *Salmonella* minnesota R595 (tlrl-smlps, InvivoGen) for 4 h and stimulated with 5 mM ATP (10127531001, Roche).

### Bacterial phagocytic uptake and proliferation assay

Phagocytosis of bacteria by macrophages was calculated by plating the macrophage cell lysates 30 min post-infection. For quantifying bacterial survival in BMDMs, cells were infected with *popB* at an MOI of 10 for 3 h. Cells were washed twice with PBS and were treated with 100 μg/ml gentamicin for 1 h to kill the extracellular bacteria. Cells were maintained in DMEM containing 10 μg/ml of gentamicin and were lysed 4- and 16-h post-infection. Bacterial numbers in cell lysates were quantified by plating cell lysates in LB agar.

### Western blot

BMDMs were lysed in RIPA buffer and sample loading buffer containing SDS and 100 mM DTT. Proteins were separated on 10–12% polyacrylamide gels. Following electrophoretic transfer of protein onto PVDF membranes, membranes were blocked in 5% skim milk and incubated with primary antibodies against caspase-1 (Adipogen, AG-20B-0042), caspase-11 (NB120-10454, Novus), anti-β-actin (#8457; Cell Signaling Technology) followed by secondary anti-rabbit or anti-mouse HRP antibodies (Jackson ImmunoResearch Laboratories).

### Immunofluorescence staining

Following infection, BMDMs were washed twice with PBS and incubated with media containing FAM FLICA caspase-1 for 1 h (ImmunoChemistry Technologies). Cells were then fixed in 4% paraformaldehyde for 15 min. Cells were incubated with a mouse anti-Asc antibody (1:500 dilution, clone 2EI-7; Millipore) overnight followed by incubation with a rabbit anti-caspase-8 (1:500 dilution, 8592; CST) for an additional 1 h. The secondary antibodies used were anti-rabbit Alexa Fluor 647 and anti-mouse Alexa Fluor 568. Cells were counterstained with DAPI. Cells and inflammasomes were visualized, counted, and imaged using a Nikon C2 confocal microscope at the Cell and Tissue Imaging Center Light Microscopy Facility (CTIC-LM) at St. Jude.

### Real time quantification of cell death

For real time quantification of cell death, BMDMs were seeded into 12 well plates for overnight incubation. Following bacterial infection, 20 nM SytoxGreen (Molecular Probes) was added, and the cells were moved into an IncuCyte live cell imaging system. Cells were imaged every 30 min and the Sytox Green labeled cells (counted as dead cells) were quantified by the IncuCyte FLR or Zoom software (http://www.essenbioscience.com/en/products/software/).

### Cytokine measurement by ELISA

IL-1β, IL-18, and IL-6 were measured using ELISA kits (eBioscience) according to the manufacturers’ instructions.

### Statistical analysis

GraphPad Prism 7 software was used for statistical analysis. Statistical significance was determined by a two-tailed *t* test. A *p* value, 0.05 was considered significant (*p < 0.05, **p < 0.01, ***p < 0.001,****p < 0.0001).

## Electronic supplementary material

Supplemental Material
