# Supplementary material for: Guanylate binding proteins facilitate caspase-11-dependent pyroptosis in response to type 3 secretion system-negative Pseudomonas aeruginosa
Source: Cell Death Discov. 2018 Jun 27;4:66. doi: 10.1038/s41420-018-0068-z (PMC6060091; doi:10.1038/s41420-018-0068-z)
Supplement: Supplementary file 1 — Supplemental Material [file 41420_2018_68_MOESM1_ESM.docx]

**Supplementary Information**

**Guanylate binding proteins facilitate caspase-11-dependent pyroptosis in response to type 3 secretion system-negative *Pseudomonas aeruginosa*.**

Arjun Balakrishnan^1^, Rajendra Karki^1^, Brent Berwin^2^, Masahiro Yamamoto^3^, Thirumala-Devi Kanneganti^1*^.

^1^*Department of Immunology, St. Jude Children's Research Hospital, Memphis, TN, 38105.*

^2^*Department of Microbiology and Immunology, Geisel School of Medicine at Dartmouth, Lebanon, NH, 03756 USA.*

^3^*Department of Immunoparasitology, Research Institute for Microbial Diseases, Laboratory of Immunoparasitology, World Premier International Immunology Frontier Research Center, Osaka University, 3-1 Yamadaoka, Suita, Osaka 565-0871, Japan.*

**Figure S1:** Inflammasome activation in BMDMs infected with indicated strains of *P. aeruginosa*. A-C) Immunoblot analysis of caspase-1, IL-1β and IL-18 release in unprimed BMDMs left untreated or infected with indicated strains of *P. aeruginosa* (MOI 10) for 2h. Data are representative of three independent experiments. ns-not significant, *p < 0.05, **p < 0.01 (two tailed T test). D) Immunoblot analysis of caspase-1 in WT, *Gbp2^-/-^*, *Gbp5^-/-^*, *Gbp^chr3^-KO* , *Irgb10^-/-^* , *Irgb10^-/-^Gbp^chr3^-KO*, *Caspase11^-/-^* and *Nlrp3^-/-^* BMDMs infected with *P. aeruginosa* PA14 (MOI10) for 2h.

**Figure S2:** *P. aeruginosa* activates NLRP3 and NLRC4 to form  leads a single ASC complex in macrophages. A) WT, *Nlrc4^-/-^*,*Nlrp3^-/-^* and *Nlrp3^-/-^Nlrc4^-/-^* unprimed BMDMs were infected with of *P. aeruginosa* PA14 (MOI 10) for 1h and stained for ASC (red), active caspase-1 (green), caspase-8 (magenta) and DNA (blue). Arrow heads indicate an inflammasome complex. B) Composition of ASC specks. At least 200 BMDMs infected with indicated strains were counted. C) Immunoblot analysis of caspase-1 in WT, *Ripk3^-/-^* and *Ripk3^-/-^Cas8^-/-^* unprimed BMDMs infected with *P. aeruginosa* PA14 (MOI 10) for 2h. D) Immunoblot analysis of caspase1 in WT, *Nlrp3^-/-^Nlrc4^-/-^*, *Asc^-/-^* and *Caspase1^-/-^* unprimed BMDMs infected with *P. aeruginosa* PA14 (MOI 10) for 2h. Data are representative of two independent experiments.

**Figure S3: Quantification of cell death during *popB* infection.**

**A,B)** Real time quantification of cell death (Sytox green incorporation) in WT, *Gbp2^-/-^, Gbp5^-/-^, Gbp^chr3^*-KO*, Irgb10^-/-^ , Irgb10^-/-^Gbp^chr3^*-KO, *Caspase11^-/-^* and *Nlrp3^-/-^*  BMDMs infected with *popB* (MOI 10). (Scale,200mm)
